# Threat Sensitivity, Stress, and Alcohol Use in Probable PTSD: Findings from a Mobile Attention Bias Modification Trial

**DOI:** 10.1007/s10608-026-10751-0

**Published:** 2026-05-30

**Authors:** Audrey Morrow, Mikael Rubin, Andrea Niles, Paige Tripp, Derek D. Satre, Joshua D. Woolley, Aoife O’Donovan, Kristen Nishimi

**Affiliations:** 1 University of California, San Francisco, USA; 2 San Francisco VA Health Care System, San Francisco, USA; 3 Palo Alto University, Palo Alto, USA; 4 Division of Research, Kaiser Permanente, Oakland, USA

**Keywords:** Posttraumatic stress disorder, PTSD, Threat sensitivity, Attention bias, Avoidance, Hypervigilance, Alcohol consumption

## Abstract

**Purpose:**

Posttraumatic stress disorder (PTSD) is characterized by heightened threat sensitivity, including attentional bias to threat, and associated with increased alcohol consumption. Given that alcohol consumption may be driven by or worsen PTSD symptoms, it is critical to consider how threat sensitivity relates to drinking in individuals with probable PTSD.

**Methods:**

In this study, 687 adults with probable PTSD (PTSD Checklist for DSM-5 symptoms ≥ 33; M_age_=30.01; 80% female) completed a 15-day app-based attention bias modification (ABM) protocol to reduce threat-related biases. Participants were randomized into personalized ABM, non-personalized ABM, or placebo training (days 2–7, 9–14), and completed dot-probe detection assessments on days 1, 8, and 15. Participants reported alcoholic drinks consumed in the past 24 h every training day. We examined associations of ABM condition, threat sensitivity (self-reported, avoidance and vigilance reaction time biases), stress (past, anticipated), and PTSD symptoms with alcohol consumption (drink count, heavy drinking) using mixed models.

**Results:**

Personalized ABM was not significantly associated with reduced drink count, relative to placebo (rate ratio *[RR]* = 0.72, 95% CI 0.51, 1.01). Higher self-reported threat sensitivity was associated with lower next-day drink count (*RR* = 0.93, 95% CI 0.87, 0.99; marginal after multiple corrections), while there was a non-significant effect between avoidance bias and lower next-day drink count (*RR* = 0.88, 95% CI 0.76, 1.02). Higher past and anticipated stress were associated with lower next-day drink count (*RR* = 0.92, 95% CI 0.87, 0.96) and heavy drinking (odds ratio *[OR]* = 0.91, 95% CI 0.82, 1.00), respectively.

**Conclusions:**

Our findings link threat sensitivity and stress with subsequent lower alcohol consumption, necessitating further research on the intersection of these factors with alcohol use in populations with probable PTSD.

**Electronic supplementary material:**

The online version of this article (https://doi.org/10.1007/s10608-026-10751-0) contains supplementary material, which is available to authorized users.

## Introduction

Posttraumatic stress disorder (PTSD) is a debilitating psychiatric condition characterized by high levels of distress and changes in physiological and affective responses to threat following a traumatic event (Kessler et al., 2017). Such changes can give rise to a state of heightened threat sensitivity, defined as a condition in which perceptual, emotional, physiological, and behavioral systems are primed to detect, appraise, respond to, and encode potentially threatening information with greater reactivity (O’Donovan et al., 2013). Notably, threat sensitivity can manifest as being avoidant of or hypervigilant towards threatening stimuli, with avoidance and hypervigilance to threat representing subdomains of threat sensitivity more broadly (Badura-Brack et al., 2018; Niles et al., 2018). Individuals with PTSD experience wide-ranging symptoms and often meet clinical criteria for at least one other psychiatric diagnosis, commonly alcohol use disorder (AUD; Hawn et al., 2020; Possemato et al., 2015). Additionally, up to 63% of individuals with PTSD have unhealthy alcohol use patterns, e.g., heavy episodic drinking or drinking over recommended lower-risk drinking guidelines (Debell et al., 2014). Many theories around the etiology of comorbid AUD-PTSD indicate that PTSD symptoms may be a driver of unhealthy alcohol use (Gilpin & Weiner, 2017; McCauley et al., 2012). Given that threat sensitivity is thought to underlie several core PTSD symptoms (Niles et al., 2020), it is critical to understand the association between threat sensitivity and alcohol use patterns.

A large body of research has focused on better understanding and treating AUD-PTSD comorbidities (Allen et al., 2016; Flanagan et al., 2016; McCauley et al., 2012; Petrakis & Simpson, 2017; Torchalla et al., 2012; Tripp et al., 2019; van Dam et al., 2012; van Minnen et al., 2015), yet there are many challenges associated with integrated treatments for AUD and PTSD, such as high attrition rates (Flanagan et al., 2016; Szafranski et al., 2019; van Dam et al., 2012) and participant disapproval of the treatment (Torchalla et al., 2012). While the causal relationships underlying PTSD-AUD comorbidity are not fully explicated, a bidirectional relationship is likely. The social-cognitive model and self-medication hypothesis suggest that alcohol use is a learned response to stress (Alexander & Ward, 2018; Bandura, 2000), and is used to alleviate symptoms (Hawn et al., 2020; Khantzian, 1997). More research is needed to understand the links between specific PTSD symptoms and alcohol use to inform better integrated interventions.

Drinking behaviors may be related to specific PTSD symptom clusters, including intrusions of trauma-related thoughts and memories; avoidance of trauma reminders; negative changes in cognition and mood; and changes in reactivity and arousal related to the trauma (Friedman et al., 2011). In particular, exaggerated threat sensitivity may manifest in symptom domains of PTSD including avoidance, specifically avoidance of internal and external threat reminders, and arousal/reactivity, which includes self-reported hypervigilance and exaggerated threat responses. Behaviorally, individuals with PTSD tend to avoid experiences and reward in the context of threat, demonstrating avoidance (Pickett et al., 2011; Weaver et al., 2020). Additionally, individuals with PTSD exhibit increased startle responses and impulsivity in response to threat stimuli, reflective of hypervigilance (Grillon et al., 1998; Niles et al., 2018; Young et al., 2020). Importantly, individuals with PTSD can experience both avoidance of and hypervigilance to threat, with some oscillating between these states (Kimble & Hyatt, 2019; Mogg et al., 2004; Pineles et al., 2011). Studies have found that avoidance behaviors are linked with alcohol use or the development of AUD in individuals with PTSD (Dvorak et al., 2013; Feingold & Zerach, 2021; Flynn et al., 2022), and hyperarousal symptoms have been found to be more prevalent in individuals with comorbid PTSD and AUD (Saladin et al., 1995). Although there is extensive work examining behavioral attentional biases (to alcohol-related cues in particular) and effects of ABM in AUD (Bollen et al., 2021, 2024; Heitmann et al., 2018), it is unclear how avoidance and hypervigilance components of threat sensitivity relate to patterns of alcohol consumption among individuals with PTSD symptoms.

Avoidance and hypervigilance biases can be measured using attention bias tasks and have been reduced following attention bias modification (ABM), a cognitive training paradigm used across various clinical conditions, in some but not all studies (Martinelli et al., 2022). In brief, threat-related ABM typically involves training to direct one’s attention away from threatening and towards neutral stimuli, intending to reduce bias towards threat. The few available studies of threat-related ABM interventions for PTSD have shown largely null effects on reduction of PTSD symptoms, and there is little evidence that ABM intervention leads to changes in attentional biases (MacLeod & Clarke, 2015; Niles et al., 2020; Schoorl et al., 2012). The ABM paradigm has also been tested with substance-related cues specifically to decrease addictive behavior, with some studies showing that substance-relevant ABM lowered attentional bias to alcohol, but less reliable evidence of changes in outcomes such as alcohol consumption (Heitmann et al., 2018). Inconsistencies with prior findings across clinical condition may be due in part to the challenge of quantifying attention bias using reaction time measures, which previously showed limited reliability as traditionally scored (Chapman et al., 2019; Rubin et al., 2024). However, recent work using a more reliable quantification separating avoidance and hypervigilance biases indicates promise for threat-related probe detection tasks as a means to derive reliable attention bias scores (Rubin et al., 2024). Taken together, though its effectiveness in decreasing clinical symptoms is still unclear, ABM interventions with probe detection tasks provide an effective method to measure behavioral attentional biases.

Despite evidence that threat sensitivity underlies core PTSD symptom domains associated with alcohol consumption, and the potential of the probe detection task to provide a reaction time index of the threat-related bias component of threat sensitivity, the relationship between threat sensitivity and drinking behaviors in PTSD remains poorly understood. Moreover, no study has examined whether threat-related ABM is associated with drinking behavior among individuals with PTSD. Thus, this study had two main aims: (1) to assess whether a 15-day threat-related ABM intervention was associated with reduced alcohol consumption; and (2) to determine if threat-sensitivity, operationalized as self-reported threat sensitivity and response-based avoidance and hypervigilance bias scores, is associated with alcohol consumption among individuals with probable PTSD. Our implementation of response-based bias measures allows not only for greater reliability and specificity, but also produces scores that have previously correlated with PTSD symptom clusters, suggesting congruence with the self-reported measures (Rubin et al., 2024). Additionally, this multi-day intervention with daily assessments of drinking behavior enabled evaluation of these relationships dynamically over time. Beyond the primary aims, acute stress perceptions are related to but distinct from domains of threat sensitivity and may serve as proximal triggers for drinking in PTSD (Simpson et al., 2014). Accordingly, we included exploratory models examining perceived past and anticipated stress and PTSD symptoms as additional psychosocial predictors of drinking behavior over time.

## Method

### Design and Procedures

We performed a secondary analysis of data collected from a randomized controlled trial (RCT) of entirely remote app-based ABM versus placebo for PTSD, previously analyzed and reported by Niles et al. (2020). Participants were randomly assigned to one of three 15-day training conditions, matched by sex and PTSD symptom severity (PTSD Checklist for DSM-5 [PCL-5]): personalized ABM, non-personalized ABM, and placebo. The study was approved by the Institutional Review Board at the University of California, San Francisco. For additional information, see Niles et al. (2020).

Participants had 21 days to complete study procedures, which included an introductory day, 3 days of attention bias testing, and 12 days of ABM training. Baseline PTSD symptoms were collected on the introductory day (Day 0). On days 1, 8, and 15, participants completed a threat-related dot probe detection task to assess attentional bias and the PCL-5. On days 2–7 and 9–14, participants completed attention training and self-reported threat sensitivity, perceived stress, and alcohol consumption.

### Participants

Participants were recruited through Reddit and Craigslist. Recruitment and data collection were conducted between January 2018 and October 2018. Interested individuals completed a screening questionnaire on Qualtrics and were determined eligible if they were over 18 years old, scored 33 or above on the PCL-5 (i.e., meeting criteria for probable PTSD (Blevins et al., 2015), and did not report active suicidal ideation. Notably, there was no explicit assessment of DSM-5 Criterion A trauma exposure or formal diagnosis of PTSD for participants. Of the 1001 individuals who were both eligible and interested in participating, 687 completed the baseline assessment on the mobile app and were included in the analysis. The study had a high retention rate, with roughly 75% of participants completing at least 10 of the 12 training sessions.

### Attention Training Conditions

The ABM training conditions (personalized, non-personalized, and neutral) varied only in the word stimuli. In all conditions, participants were asked to maintain their gaze on a central fixation and respond according to which letter probe appeared on the trial. On each trial, a fixation cross was presented for 500ms, followed by a pair of word stimuli that appeared above and below fixation, 3 cm apart. The stimulus sizes were programmed to remain constant across different phone screen sizes and should have occupied 0.5–1.5 degrees of visual angle, depending on how participants held their phone. The word stimuli remained on the screen for 500ms, and a probe letter (“E” or “F”) appeared in place of one of the words.

The word selection for the personalized condition used a recommender (Niles & O’Donovan, 2018) to select 60 words from a pool of 453 threat words that were not used in the initial threat assessment. The initial threat assessment occurred upon opening the app and asked participants to rate threat words on a Likert scale from 1 (most threatening) to 9 (least threatening). The recommender system identified participants who rated threat words similarly and then used these initial ratings to estimate how each participant in the personalized condition might rate the remaining 453 words. The 60 words that had the highest estimated ratings for each participant were used in the personalized condition.

In the non-personalized condition, threat words were randomly selected from the pool of words that had an average rating of 4 or higher in the initial threat assessment. All participants in the non-personalized condition saw the same 60 threat words.

In the placebo condition, both words were neutral on all trials. In the personalized and non-personalized conditions, threat words could appear randomly above or below the central fixation, opposite the neutral word. The probe type and location and the threat word location were counterbalanced and randomized across trials, and the probe appeared in place of the neutral (vs. threat) word every time. The probe remained on screen until participants input their response using the corresponding letter keys. Participants in all conditions completed 90 trials on each training day.

### Measures

#### Threat Sensitivity

 We included multiple measures of threat sensitivity, encompassing different subdomains (e.g., perceived threat, vigilance, avoidance) and modalities (i.e., self-report, attentional bias reaction times).

*Threat Sensitivity Scale (TSS)*. The TSS was an 8-question scale that asked participants to rate self-reported aspects of threat sensitivity over the past 24 h. This scale was developed for use in Niles et al. (2020) and consisted of items from different scales to measure perceived threat sensitivity specifically related to perceived attentional control, perceptions of acute threat, and perceived hypervigilance. These included three items from the Attentional Control Scale (Derryberry & Reed, 2002), three items from the Threat Orientation Scale (Thompson et al., 2006), and two items from the Arousal/Reactivity subscale of the PCL-5 (Blevins et al., 2015). The scale included prompts such as, “I was jumpy and easily startled,” and “I felt I was more at risk than I actually was.” Responses were provided on a 5-point Likert scale ranging from “not at all” to “extremely” and all 8 items were summed to create total TSS scores for each day. TSS sum scores had high internal consistency reliability (Cronbach’s alpha range 0.925–0.931 across training days).

*Avoidance and Hypervigilance Reaction Time (RT) Biases*. Threat sensitivity was also assessed using the dot probe detection task to derive reaction time behavioral measures of avoidance and hypervigilance biases. In the dot probe detection task, participants engaged in a variation of the ABM training protocol whereby the probe replaced threat words 50% of the time. Trials where the probe letter followed the threat stimulus were considered congruent trials, and trials where the probe letter followed the neutral stimulus were considered incongruent. Threat word location, probe location, and probe type were counterbalanced and randomized across trials. There were 70 trials on each dot probe detection task day.

Avoidance and hypervigilance RT biases were derived using the trial-level response-based attention bias RB-AB computation (Evans & Britton, 2018). The mean RT for all congruent trials was computed for each participant, and then individual incongruent trial RTs were subtracted from the mean congruent RT, providing trial-level difference scores. Negative trial-level difference scores indicated avoidance of the threat stimulus (“Avoidance Bias”), while positive trial-level difference scores indicated hypervigilance towards the threat stimulus (“Hypervigilance Bias”). We computed the mean of the negative and positive RT differences separately, such that each participant had separate overall avoidance and hypervigilance bias scores on each dot probe detection task day (Rubin et al., 2024). Incorrect trials and any trials where the RTs were faster than 150ms or slower than 2000ms were removed prior to analysis. Additionally, we removed participants with fewer than 70% of trials remaining after data cleaning, leaving 658 participants for RB-AB analyses.

#### Alcohol Consumption

 Participants were asked their amount of recent alcohol consumption on each training day. Participants were asked, “How many alcoholic drinks did you have in the past 24 h?” and could select from the following options: 0 drinks, 1–2 drinks, 3–5 drinks, 6–10 drinks, 10 or more drinks. Drinking data was used in two ways: to indicate daily drink count and whether participants had engaged in heavy drinking on a given day. Drink count was quantified as the midpoint of each range, rounding down to the nearest integer. We extrapolated from the increasing ranges of each drinking category that 10 or more drinks would range from 10 to 18 drinks. Therefore, drink count was defined as 0 (0 drinks), 1 (1–2 drinks), 4 (3–5 drinks), 8 (6–10 drinks), and 14 (10 or more drinks). Heavy drinking was quantified as a binary variable, similar to National Institute on Alcohol Abuse and Alcoholism guidelines of heavy drinking as 4 or more drinks for females or 5 or more drinks for males on one occasion (National Institutes of Health, 2026, Jan). All responses indicating 3–5 drinks and above were coded as heavy drinking, providing a conservative estimate of heavy drinking patterns.

#### Stress

 Given potential relationships between day-to-day stress and alcohol consumption, we collected stress ratings from the past 24 h (“past stress”) and anticipated for the next 24 h (“anticipated stress”) on each training day. For past stress, participants were asked, “How stressful have the past 24 h been?”, and for anticipated stress, “How worried are you about the next 24 h?”. Both questions were answered using a 5-point Likert scale from “not at all” to “extremely”. These single-item measures were developed for the current ABM study, though are similar to other ecological momentary assessment study assessments of subjective stress with adequate validity (Hojjaty & Zawadzki, 2024; Weber et al., 2022).

#### PTSD Symptoms

 Participants completed the PCL-5 on days 1, 8, and 15 (Blevins et al., 2015). The measure includes 20 questions designed to address the 20 symptoms associated with PTSD according to the *Diagnostic and Statistical Manual of Mental Disorders* (DSM–5, American Psychiatric Association, 2013). Participants rated how much they were bothered by each symptom in the past month, according to a 5-point Likert scale from “not at all” to “extremely”. A total symptom score was calculated by summing responses across all scale items for each day. The PCL-5 showed high internal consistency reliability (Cronbach’s alpha range 0.936–0.944 across days).

### Statistical Analyses

We assessed the relationship between ABM treatment condition assignment and alcohol consumption as well as threat sensitivity measures and alcohol consumption over time. To examine whether ABM treatment conditions were associated with drinking, we used a generalized linear mixed effects (GLME; using MATLAB function fitglme) model with ABM condition predicting drinking on days 2–7 and 9–14. Models adjusted for study day to account for time across the study and sex (male or female) as a potential confounder. The models included a random intercept for participant and random slope for study day, which provided a better fit compared to models without a random slope. We modeled drink count and heavy drinking as separate outcomes. For drink count, we used a Poisson-distribution GLME model with a log link predicting count of drinks to derive rate ratios (RRs). Examining model output, we determined that there was no overdispersion in Poisson GLME models with drink count. For heavy drinking, we used a Binary-distribution GLME model with a logit link predicting the odds of engaging in heavy drinking to derive odds ratios (ORs).

Similar GLME models were conducted separately for each threat sensitivity measure (i.e., TSS, avoidance, hypervigilance) as the predictor of interest, with the predictors being time-updated. We structured the data such that threat sensitivity measures were aligned with drinking on the immediately subsequent day of the study (e.g., TSS from day 2 was aligned with drinking from day 3). The one-day lag was intended to capture threat sensitivity and other symptom effects on subsequent drinking behavior the next day, to avoid reverse causality by contemporaneous reporting (e.g., current day’s drinking influencing concurrently reported symptoms). All models included ABM condition, study day, and sex as covariates with random intercepts and random slopes for study day. All continuous predictors were z-scored. We also performed both the drink count and heavy drinking models with exploratory predictors of interest: Past Stress, Anticipated Stress, and PTSD symptoms.

While we did not have any hypotheses about study day or sex as moderators of the relationship between predictors and drinking, we tested interactions in exploratory models. Across all fourteen models, we found only one significant interaction between study day and predictor variables (past stress and drink count), and one between sex and predictor variables (anticipated stress and drink count). Interaction terms for study day and sex were thus not included in our main models to keep the models consistent and focus on our main hypotheses.

Our primary confirmatory analyses were (1) ABM condition predicting alcohol consumption and (2) threat sensitivity predicting alcohol consumption. ABM models included a single pre-specified test per outcome, thus raw, uncorrected p-values were reported. Threat sensitivity was assessed with three measures as a family of tests operationalizing subdomains of a common construct, thus Benjamini-Hochberg false discovery rate (BH-FDR) correction was applied for the three measures separately by outcome; uncorrected and BH-FDR corrected *p*-values were reported. All other analyses were considered exploratory and hypothesis generating, thus, consistent with recommendations, we did not apply p-value corrections but results should be interpreted as preliminary requiring independent replication (Rothman, 1990).

We conducted sensitivity analyses to examine whether effects were impacted by abstinence from alcohol. We removed participants who reported not drinking throughout the entire study, then conducted all previously described models among the remaining subsample (*n* = 347 who reported drinking at least once during the study). The reasons for this were twofold: (1) there was a high proportion of “0 drinks” responses each day (Supplemental Fig. 1) and, importantly, 54% of participants did not ever report drinking, making the data skewed towards 0, and (2) studies have indicated that individuals who do not drink show different health behaviors than individuals who have occasional low numbers of alcoholic beverages (Evans-Polce et al., 2016; Rosansky & Rosenberg, 2020).

## Results

The sample (*n* = 687) had an age range of 18–69 years old (M = 30.1; SD = 10.0), was 80.2% female, and 60.0% White, 12.9% Hispanic, 12.0% Black, 8.3% Asian, and 8.4% other/more than one race. Participants had a mean baseline PCL-5 score of 45.5 (SD = 12.1), which is considered in the clinical range (scores 33+) for a probable PTSD diagnosis, and a mean score of 37.3 at the end of the study. There was an average of 0.6 drinks per day (SD = 1.7) reported among the sample across the study (daily frequencies are shown in Supplemental Fig. 1). On the first training day, 75.2% reported no drinks, 15.8% reported 1–2 drinks, 7.2% reported 3–5 drinks, 2.8% reported 6–10 drinks, and 0.5% reported more than 10 drinks, whereas on the final training day, 80.5% reported no drinks, 11.1% reported 1–2 drinks, 5.5% reported 3–5 drinks, 2.1% reported 6–10 drinks, and 0.2% reported more than 10 drinks. Participants, on average, reported heavy drinking on 21.5% of days.

Among 687 total participants, 211 (30.7%) had some missing data on at least one day, and 109 (15.9%) participants had some missing data from at least half of the study days. However, when considering all possible days and items from measures of interest across all participants, the total missingness of items was very low at 0.29% and item-level missingness of predictor variables was < 0.4%. RT data that contributed to the computations for avoidance and hypervigilance biases was missing 13.6% of trials. Importantly, GLMEs incorporate all available data and can provide unbiased estimates even without complete datasets.

## ABM and Alcohol Consumption

**Fig. 1:**
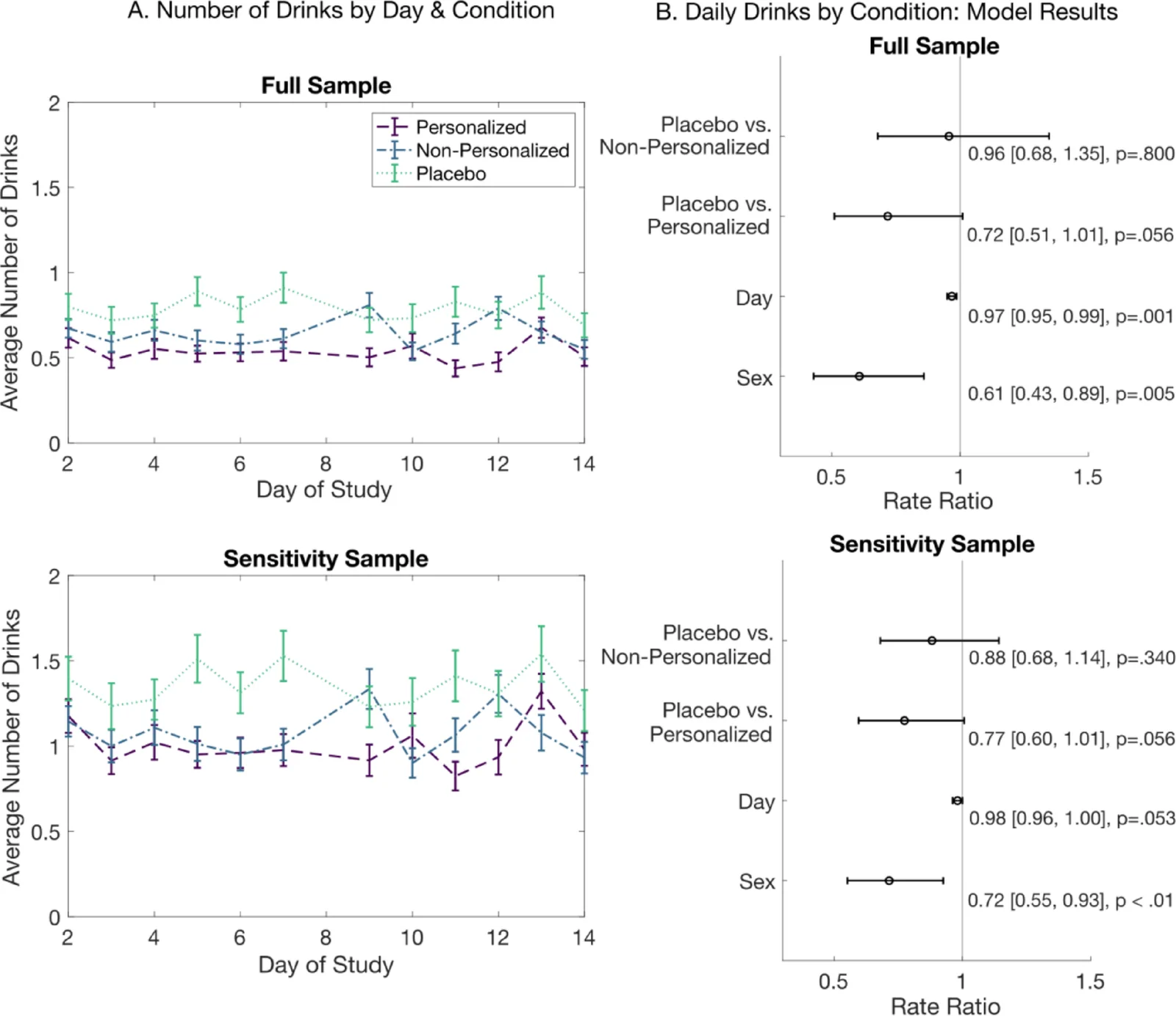
Associations between Attention Bias Modification (ABM) condition and daily drink count. **A** The mean number of alcoholic drinks participants reported consuming by study day and condition are plotted for the full sample of participants (top; n = 687) and the sensitivity sample (bottom, n = 347), which excluded participants who did not drink on any day of the study. **B** Results from the general linear mixed effects models predicting the number of drinks participants reported consuming during the study, predicted by study day, sex (male as reference), and condition for the full sample of participants (top) and the sensitivity sample (bottom).

ABM condition was not significantly associated with drinking behaviors (personalized vs placebo *RR* = 0.72, 95% CI 0.51, 1.01, *p*=0.056) (Fig. 1), corresponding to approximately 0.25 fewer drinks per day. The non-personalized ABM condition did not differ from placebo (*RR* = 0.96, 95% CI 0.68, 1.35, *p*=0.800). Baseline drink count did not differ between any conditions (T-tests for drink count by each group comparison: placebo vs. personalized: *t*=−0.84, *p*=.40, placebo vs. non-personalized: *t*=−0.32, *p*=0.75, personalized vs. non-personalized: *t*=−0.53, *p*=0.59). We found a significant effect of study day (*RR* = 0.97, 95% CI 0.95, 0.99, *p*=0.001), such that participants drank less on average as the study progressed. Additionally, we found a significant association between sex and drink count (*RR* = 0.61, 95% CI 0.43, 0.89, *p*=0.005), such that female participants drank significantly less than male participants. We found no association between ABM condition and heavy drinking (Supplemental Table 2), though similar to drink count models, heavy drinking decreased over the course of the study (*OR* = 0.97, 95% CI 0.95, 0.99, *p*=0.005) and females had less heavy drinking than males (*OR* = 0.55, 95% CI 0.37, 0.81, *p*=0.003).

## Threat Sensitivity and Alcohol Consumption

Higher TSS scores were associated with lower drink count on subsequent days (*RR* = 0.93, 95% CI 0.87, 0.99, *p*=0.020, *p*_*BH−FDR*_=0.06), corresponding to approximately 0.07 fewer drinks per each standard deviation higher TSS score. Notably, this association did not retain statistical significance (*p*<0.05) after multiple corrections, and should be interpreted cautiously. Avoidance bias was not significantly associated with drink count (*RR* = 0.88, 95% CI 0.76, 1.02, *p*=0.081, *p*_*BH−FDR*_=0.12; Table 1). Hypervigilance bias was not associated with drink count, and none of the threat sensitivity measures were associated with heavy drinking (Supplemental Table 3).

**Table 1:** Associations of Attention Bias Modification (ABM) training, threat sensitivity, and other symptom measures with daily drink count, among the full sample (*n* = 687) and sensitivity subsample excluding non-drinkers (*n* = 347)

	Outcome: daily drink count	
	Full sample	Sensitivity subsample
Primary predictor	Rate ratio [95% CI]	Rate ratio [95% CI]
*ABM condition*		
Personalized	*0.72 [0.51, 1.01]+*	*0.77 [0.60, 1.01]+*
Non-personalized	0.96 [0.68, 1.35]	0.88 [0.68, 1.14]
Placebo	Ref	Ref
*Threat sensitivity measures*		
Threat sensitivity scale (TSS; self-report)	0.93 [0.87, 0.99]*†	*0.94 [0.89*,* 1.00]+*
Avoidance bias (reaction time)	*0.88 [0.76*,* 1.02]+*	0.96 [0.84, 1.10]
Hypervigilance bias (reaction time)	1.01 [0.88, 1.16]	1.05 [0.92, 1.21]
*Other symptom measures*		
Past stress	0.92 [0.87, 0.96]**	0.92 [0.88, 0.96]**
Anticipated stress	0.99 [0.94, 1.04]	0.99 [0.94, 1.04]

Model results from separate Poisson mixed effects models with the primary predictor and daily drink count outcome, adjusting for ABM condition (personalized, non-personalized, placebo), study day, and sex. Rate Ratios represent the relative rate that a predictor is associated with a unit change in the number of drinks consumed on subsequent study days. Significance is indicated after confidence intervals: *+p*_raw_<0.10, *p_raw_<0.05, **p_raw_<0.01.

BH-FDR corrected *p*-values (applied to threat sensitivity models): †p_BH−FDR_<0.10, ††p_BH−FDR_<0.05.

### Secondary Models

Analysis of exploratory predictors (Table 1; Supplemental Table 3) showed that higher past stress was associated with lower drink count (*RR* = 0.92, 95% CI 0.87, 0.96, *p*<0.001), but not with heavy drinking. The association between anticipated stress and odds of heavy drinking (*OR* = 0.91, 95% CI 0.82, 1.00, *p*=0.054) was not significant. No relationship was found between PTSD symptoms and either measure of alcohol consumption.

We found a significant interaction of day and sex as predictors in two models. There was a significant interaction between study day and past stress on the effect of drink count (*RR*_*interaction*_=1.02, 95% CI 1.01, 1.03, *p*=0.004) such that the strength of the association between past stress and next-day drink count decreased slightly over time. There was also a significant interaction between sex and anticipated stress for drink count (*RR*_*interaction*_=0.81, 95% CI 0.71, 0.92, *p*=0.001); in the sex interaction model, higher anticipated stress was associated with higher next-day drink count, but the association was attenuated (19% lower) for female compared to male participants.

Sensitivity analyses excluding non-drinking participants showed a similar pattern of results (Fig. 1; Table 1). Personalized ABM condition, relative to placebo, was not significantly associated with lower drink count (*RR* = 0.77, 95% CI 0.60, 1.01, *p*=0.056) and was not significantly associated with heavy drinking. TSS remained associated with lower drink count (*RR* = 0.94, 95% CI 0.89, 1.00, *p*=0.040), as did past stress (*RR* = 0.92, 95% CI 0.88, 0.96, *p*<0.001). Anticipated stress was associated with lower odds of heavy drinking (*OR* = 0.90, 95% CI 0.82, 1.00, *p*=0.040). However, there was no longer a relationship between avoidance and drink count when excluding non-drinkers.

## Discussion

Using repeated measures of threat-related symptoms and drinking across 15 days of assessment, this study evaluated relationships of ABM, threat sensitivity, and stress with alcohol use among individuals with probable PTSD. ABM condition was not significantly associated with drinking. Surprisingly, we also found that greater threat sensitivity (self-reported TSS) was associated with fewer next-day drinks and that stress from the past day and anticipated stress of the upcoming day were associated with reduced heavy drinking and number of drinks, respectively. Notably, the study did not assess or restrict based on AUD status, and participants reflected a broad range of alcohol consumption from non-drinkers to heavy drinkers. Sensitivity analyses removing non-drinking participants showed a similar pattern of findings. Overall, results suggest that higher levels of several PTSD-related measures were linked with lower subsequent alcohol consumption, despite the fact that PTSD symptoms themselves were not associated with subsequent alcohol consumption.

The mixed findings regarding the relationship between personalized ABM and lower drink count across the 15-day paradigm indicates the need for more research on the potential application of ABM as an intervention for drinking behaviors among individuals with probable PTSD. The personalized condition (compared to placebo) was significantly associated with lower drinking when adjusting for TSS, past stress, and anticipated stress. However, we did not find an interaction between ABM and study day, suggesting that the amount participants drank did not change over time differentially across study groups. Thus, it is not clear how much of the association is due to chance assignment, despite no differences in baseline drinking, or due to changes in behavior from ABM. Additional studies are needed to confirm these associations, explore different forms of ABM, and, generally, better understand the mechanisms underlying this possible relationship. We speculate that the personalized stimuli had a greater impact on participants, potentially leading to reduced drinking behaviors, but our findings do not support a causal relationship between personalized ABM and alcohol consumption.

The associations between higher threat sensitivity measures and lower drinking are inconsistent with the self-medication and social-cognitive models (McCauley et al., 2012), which suggest alcohol is used to reduce negative symptoms. Importantly, these associations with lower drinking were specific to self-reported threat sensitivity and avoidance bias, but may not represent robust or replicable effects. These findings could highlight a unique pattern of alcohol consumption in a subset of individuals with probable PTSD, such as those with higher avoidance. This idea is partially supported by an ecological momentary assessment of drinking in combat veterans with PTSD, in which greater PTSD symptomatology was associated with a same-day increase in drinking, but measures of avoidance coping and self-efficacy to resist drinking significantly moderated this association and predicted a reduction in later drinking (Possemato et al., 2015). It is possible that heightened perceived threat reactivity and avoidance could lead to withdrawal and social avoidance (Gellner et al., 2020), thus reducing opportunities for social drinking. A related important construct may be attentional control, or the capacity to voluntarily regulate and direct attention (Derryberry & Reed, 2002). Self-reported attentional control is not consistently associated with task-based attentional biases (Khodami et al., 2024), potentially due to indexing distinct domains of perceived regulatory capacity versus automatic processes. However, much of the prior work using task-based attention bias measures also used unreliable metrics (Chapman et al., 2019). In contrast, self-reported threat sensitivity includes items related to attentional control, though the extent to which individual differences in attentional control specifically influence observed associations with lower drinking remains unknown and represents interesting avenues for future work. Without better contextual information around drinking behaviors, it is difficult to characterize how these threat sensitivity indicators could be mechanistically related to drinking behaviors.

Notably, hypervigilance bias was unassociated with alcohol consumption. Interestingly, a prior study of 12-week combined medication and cognitive behavioral therapy for comorbid AUD-PTSD found that, while hyperarousal domain symptoms were significantly related to the response to treatment and reduced daily drinking, this domain showed the slowest and least improvement relative to avoidance and intrusion domains (Back et al., 2006). It is possible that more time or greater sensitivity is needed for relationships between drinking patterns and certain symptom clusters like hyperarousal or hypervigilance to emerge. Similarly, while our sample showed a reduction in overall PTSD symptoms by the end of the study (Niles et al., 2020), PTSD symptoms were unrelated to ABM condition in the prior study and were unrelated to alcohol consumption in our current analysis. Aligned with our threat sensitivity findings, we found that higher stress was associated with lower next-day drinking. Past stress was associated with lower next-day drinking both in the main model and the exploratory model with day as a moderator. The interaction suggested that the association between past stress and drink count weakened as the study went on, such that participants may have abstained less following subsequent stressful days.

Both stress measures were related to a reduction in next-day drinking, although anticipated stress was uniquely associated with lower heavy drinking. These findings are not consistent with the hypotheses or prior literature, which have shown greater stress predicts increased alcohol consumption (Grzywacz & Almeida, 2008; Wolkowicz et al., 2022). It is possible that the anticipated stress reported did not always match the actual stress of the next day, or that the anticipation was skewed by prior experienced stress, potentially introducing a bias not seen in the other self-report measures. However, this is speculative and a large review of binge drinking research suggests an array of determinants of heavy drinking, from personality traits to social and environmental factors, highlighting that it is complicated to predict (Kuntsche et al., 2017). It is also possible that past stress is related to more immediate or concurrent drinking, whereas our study was designed to look at longer-term associations with drinking behaviors. Overall, it seems that in our sample of individuals with probable PTSD, increases in both past and anticipated stress were not managed by next-day alcohol consumption, but rather the opposite, suggesting that stress may be a correlate of drinking behaviors in a population with PTSD as well as an area for future research to better understand how stress relates to PTSD symptom management.

Interestingly, the relationship between anticipated stress and drink count differed by sex. Specifically, higher anticipated stress was significantly associated with higher drinking on subsequent days among males, but was unassociated with drinking in females. While this interaction was exploratory and did not appear in any other models, it highlights the need for further exploration of sex differences in PTSD symptoms and behavior. Prior research of related sex differences is mixed; for example, a study of individuals with comorbid AUD-PTSD found men and women are equally likely to drink to cope, but men were more likely to drink to enhance positive feelings (Lehavot et al., 2014), while another study found that college-aged women with PTSD were more likely to drink when experiencing higher feelings of positive affect (Kaysen et al., 2014). There are also potential sex differences in self-medication with alcohol use (e.g., men may be more likely to use alcohol to lower hyperarousal symptoms and women to reduce negative affect (Hoopsick et al., 2026; Peltier et al., 2019)), reflecting the male-specific anticipated stress-drinking patterns we identified. In combination, these findings point to potentially nuanced ways that sex relates to alcohol consumption and stress that may be moderated by comorbid diagnoses.

### Limitations

There were several limitations in our ability to address the specific relationship between threat sensitivity and alcohol consumption. We identified several non-significant effects, where the p-values of the associations may reflect an underpowered sample. Non-significant findings cannot be interpreted directly, however the effect sizes observed may provide opportunities for optimizing power and analysis design in other samples. The drinking measure did not assess exact quantities of alcoholic drinks consumed per day or timing of drinks. This impacted our ability to accurately measure heavy drinking especially, which definitionally varies by sex, and our conservative measure may have limited our ability to detect associations. Additionally, we did not collect data on whether participants had a diagnosis of AUD. While we focused broadly on patterns of alcohol use, there may be differences in how alcohol use relates to PTSD symptoms and threat sensitivity among those with and without AUD, and these data could have provided an important covariate. Although ABM condition was randomly assigned and thus any confounding from baseline factors was unlikely, our observational associations of threat sensitivity and other symptom measures with drinking may have had residual confounding. The self-reported threat sensitivity measure was created for the parent study and included PCL-5 hyperarousal items, thus there is overlap between self-reported threat sensitivity and PTSD symptom scores. Secondary measures of stress relied on single items, which may have greater measurement error than validated scales. We relied on a selected sample of individuals with probable PTSD symptoms without formal study assessments of Criterion A trauma exposure or PTSD diagnosis who responded to ads for a remote app-based intervention for PTSD symptoms, thus generalizability beyond this group is potentially limited. In particular, over half of the sample reported no alcohol consumption during the study, indicating this sample of community-based individuals may not reflect other populations where PTSD symptoms and unhealthy alcohol use patterns may be more likely to co-occur. As expected for a multi-day study, there was some missingness in responses over time, though overall missingness was low, so the impact on the main associations was likely minimal. Finally, our sample was predominantly female, comprising 551 females and 137 males, reducing power for identifying specific sex differences and examining associations in males.

## Conclusions

Our results show a novel relationship between ABM, threat and stress symptoms, and alcohol use. We found that individuals with probable PTSD are generally less likely to drink when certain stress and threat sensitivity symptoms are high. Associations also seemed symptom specific, with avoidance bias and TSS associated with reduced alcohol consumption, while hypervigilance bias and overall PTSD symptoms were not related to drinking. More research is needed to understand the nuanced relationship between PTSD symptom clusters and alcohol consumption. The multi-day app-based ABM paradigm is particularly well-suited to addressing these questions, given that it can be done remotely, can collect multiple symptom measures repeatedly, and can be easily adapted to include alcohol-related stimuli. This study has highlighted the potential for this attention training paradigm to explore links between symptom measures and behaviors across time.

## Electronic Supplementary Material

Below is the link to the electronic supplementary material.

Supplementary file 1

## Data Availability

The application-based data that support the findings of this study are available with the San Francisco Department of Veterans Affairs (VA), but restrictions apply to the availability of these data, which are stored on a secured VA server are not publicly available. The data are, however, available upon request and with the permission of the VA.
