# Supplementary material for: Threat Sensitivity, Stress, and Alcohol Use in Probable PTSD: Findings from a Mobile Attention Bias Modification Trial
Source: Cognit Ther Res. 2026 May 30;50(5):948–59. doi: 10.1007/s10608-026-10751-0 (PMC13675373; doi:10.1007/s10608-026-10751-0)
Supplement: Supplementary file 1 [file 10608_2026_10751_MOESM1_ESM.docx]

Supplemental Materials

**Supplemental Figure 1.** Frequency of responses for each drinking category and training day of the study.

Histogram showing the number of participant responses per drinking category on each day of the study. Dashed lines capture the number of responses averaged across days, for the category with the corresponding color. The total number of responses decreased by the end of the study due to participant drop-out.

| **Supplemental** **Table 1.** Associations of threat sensitivity and other symptom measures with daily drink count, among the full sample (n=687) and sensitivity subsample excluding non-drinkers (n=347) including all covariate effects | | |
| --- | --- | --- |
|  | **Outcome: Daily Drink Count** | |
|  | ***Full Sample*** | ***Sensitivity Subsample*** |
| **Predictor** | *Rate Ratio [95%CI]* | *Rate Ratio [95%CI]* |
| Threat Sensitivity Measures |  |  |
| Threat Sensitivity Scale (TSS; self-report) | **0.93 [0.87, 0.99]***† | *0.94 [0.89, 1.00]+* |
| Personalized (vs. placebo) | **0.67 [0.47, 0.96]*** | **0.73 [0.55, 0.97]*** |
| Non-Personalized (vs. placebo) | 0.96 [0.67, 1.36] | 0.88 [0.67, 1.16] |
| Placebo | *Ref* | *Ref* |
| Study Day | **0.97 [0.95, 0.99]*** | 0.99 [0.97, 1.01] |
| Female (vs. male) | **0.63 [0.44, 0.90]*** | **0.73 [0.56, 0.97]*** |
|  |  |  |
| Avoidance Bias (reaction time) | *0.88 [0.76, 1.02]+* | 0.96 [0.84, 1.10] |
| Personalized (vs. placebo) | 0.89 [0.61, 1.29] | 0.92 [0.64, 1.32] |
| Non-Personalized (vs. placebo) | 1.10 [0.76, 1.59] | 1.04 [0.72, 1.49] |
| Placebo | *Ref* | *Ref* |
| Study Day | 0.98 [0.95, 1.02] | 0.98 [0.95, 1.01] |
| Female (vs. male) | **0.61 [0.43, 0.86]**** | **0.65 [0.47, 0.92]*** |
|  |  |  |
| Hypervigilance Bias (reaction time) | 1.01 [0.88, 1.16] | 1.05 [0.92, 1.21] |
| Personalized (vs. placebo) | 0.90 [0.62, 1.30] | 0.92 [0.64, 1.32] |
| Non-Personalized (vs. placebo) | 1.11 [0.76, 1.60] | 1.05 [0.74, 1.50] |
| Placebo | *Ref* | *Ref* |
| Study Day | 0.99 [0.95, 1.02] | 0.98 [0.95, 1.02] |
| Female (vs. male) | **0.60 [0.42, 0.86]**** | **0.66 [0.47, 0.92]*** |
|  |  |  |
| Other Symptom Measures |  |  |
| Past Stress | **0.92 [0.87, 0.96]**** | **0.92 [0.88, 0.96]**** |
| Personalized (vs. placebo) | **0.67 [0.47, 0.95]*** | **0.72 [0.54, 0.95]*** |
| Non-Personalized (vs. placebo) | 0.96 [0.67, 1.36] | 0.88 [0.67, 1.16] |
| Placebo | *Ref* | *Ref* |
| Study Day | **0.97 [0.95, 0.99]**** | 0.98 [0.96, 1.00] |
| Female (vs. male) | **0.63 [0.44, 0.90]*** | **0.74 [0.56, 0.97]*** |
|  |  |  |
| Anticipated Stress | 0.99 [0.94, 1.04] | 0.99 [0.94, 1.04] |
| Personalized (vs. placebo) | **0.67 [0.47, 0.95]*** | **0.73 [0.55, 0.96]*** |
| Non-Personalized (vs. placebo) | 0.96 [0.67, 1.36] | 0.88 [0.67, 1.16] |
| Placebo | *Ref* | *Ref* |
| Study Day | **0.97 [0.95, 0.99]**** | 0.99 [0.97, 1.01] |
| Female (vs. male) | **0.62 [0.43, 0.89]**** | **0.73 [0.55, 0.96]*** |
|  |  |  |
| PTSD Symptoms | 1.07 [0.92, 1.24] | 1.06 [0.92, 1.23] |
| Personalized (vs. placebo) | 0.89 [0.62, 1.29] | 0.92 [0.64, 1.31] |
| Non-Personalized (vs. placebo) | 1.10 [0.76, 1.59] | 1.04 [0.73, 1.49] |
| Placebo | *Ref* | *Ref* |
| Study Day | 0.99 [0.96, 1.03] | 0.98 [0.95, 1.02] |
| Female (vs. male) | **0.59 [0.41, 0.85]**** | **0.64 [0.45, 0.90]*** |
| *Note.* Model results from separate Poisson mixed effects models with the primary predictor and daily drink count outcome, adjusting for ABM condition (personalized, non-personalized, placebo), study day, and sex. Rate Ratios represent the relative rate that a predictor is associated with a unit change in the number of drinks consumed on subsequent study days. Significance is indicated after confidence intervals: *+p_raw_<.10*, ***p_raw_<.05**, ****p_raw_<.01**.  BH-FDR corrected p-values (applied to threat sensitivity models): †p_BH-FDR_<.10, ††p_BH-FDR_<.05. | | |

| **Supplemental** **Table 2.** Associations of Attention Bias Modification (ABM) condition and daily heavy drinking, among the full sample (n=687) and sensitivity subsample excluding non-drinkers (n=347) including all covariate effects | | |
| --- | --- | --- |
|  | **Outcome: Daily Heavy Drinking** | |
|  | ***Full Sample*** | ***Sensitivity Subsample*** |
| **Predictor** | *Odds Ratio [95%CI]* | *Odds Ratio [95%CI]* |
| ABM Condition |  |  |
| Personalized | 0.79 [0.54, 1.16] | 0.93 [0.68, 1.29] |
| Non-Personalized | 0.99 [0.68, 1.46] | 0.89 [0.64, 1.23] |
| Placebo | Ref | Ref |
| Study Day | **0.97 [0.95, 0.99]**** | **0.98 [0.96, 1.00]*** |
| Female (vs. male) | **0.55 [0.37, 0.81]**** | **0.63 [0.46, 0.87]**** |
| *Note.* Model results from a Logistic mixed effects models with the ABM condition and daily heavy drinking outcome, adjusting for study day and sex. Significance is indicated after confidence intervals: *+p<.10*, ***p<.05, and **p<.01.** | | |

| **Supplemental** **Table 3.** Associations of threat sensitivity and other symptom measures with daily drink count, among the full sample (n=687) and sensitivity subsample excluding non-drinkers (n=347) including all covariate effects | | |
| --- | --- | --- |
|  | **Outcome: Daily Heavy Drinking** | |
|  | ***Full Sample*** | ***Sensitivity Subsample*** |
| **Predictor** | *Odds Ratio [95%CI]* | *Odds Ratio [95%CI]* |
| Threat Sensitivity Measures |  |  |
| Threat Sensitivity Scale (TSS; self-report) | 0.92 [0.82, 1.03] | 0.93 [ 0.83, 1.03] |
| Personalized (vs. placebo) | 0.77 [0.52, 1.13] | 0.86 [0.61, 1.28] |
| Non-Personalized (vs. placebo) | 0.97 [0.66, 1.44] | 0.91 [0.66, 1.28] |
| Placebo | Ref | Ref |
| Study Day | **0.98 [0.95, 1.00]*** | 0.96 [0.96, 1.01] |
| Female (vs. male) | **0.58 [0.49, 0.86]**** | **0.66 [0.47, 0.93]*** |
|  |  |  |
| Avoidance Bias (reaction time) | 1.01 [0.83, 1.23] | .97 [0.78, 1.22] |
| Personalized (vs. placebo) | 0.83 [0.51, 1.35] | 0.78 [0.44, 1.36] |
| Non-Personalized (vs. placebo) | 1.01 [0.62, 1.65] | 1.00 [0.58, 1.73] |
| Placebo | Ref | Ref |
| Study Day | 0.99 [0.94, 1.05] | 1.02 [0.96, 1.08] |
| Female (vs. male) | **0.60 [0.38, 0.95]*** | 0.68 [0.40, 1.15] |
|  |  |  |
| Hypervigilance Bias (reaction time) | 1.02 [0.86, 1.23] | 1.09 [0.88, 1.35] |
| Personalized (vs. placebo) | 0.82 [0.52, 1.28] | 0.78 [0.44, 1.36] |
| Non-Personalized (vs. placebo) | 1.02 [0.65, 1.59] | 1.01 [0.59, 1.75] |
| Placebo | Ref | Ref |
| Study Day | 1.00 [0.94, 1.05] | 1.02 [0.96, 1.09] |
| Female (vs. male) | **0.60 [0.40, 0.91]*** | 0.69 [0.41, 1.16] |
|  |  |  |
| Other Symptom Measures |  |  |
| Past Stress | 0.95 [0.87, 1.05] | 0.96 [0.87, 1.05] |
| Personalized (vs. placebo) | 0.76 [0.52, 1.12] | 0.85 [0.61, 1.19] |
| Non-Personalized (vs. placebo) | 0.97 [0.66, 1.43] | 0.85 [0.61, 1.19] |
| Placebo | Ref | Ref |
| Study Day | **0.98 [0.95, 1.00]*** | 0.99 [0.96, 1.01] |
| Female (vs. male) | **0.58 [0.39, 0.85]**** | **0.66 [0.47, 0.92]*** |
|  |  |  |
| Anticipated Stress | *0.91 [0.82, 1.00]+* | **0.90 [0.82, 1.00]*** |
| Personalized (vs. placebo) | 0.77 [0.52, 1.13] | 0.86 [0.61, 1.20] |
| Non-Personalized (vs. placebo) | 0.97 [0.66, 1.43] | 0.91 [0.66, 1.27] |
| Placebo | Ref | Ref |
| Study Day | **0.98 [0.95, 1.00]*** | 0.99 [0.96, 1.01] |
| Female (vs. male) | **0.58 [0.39, 0.87]**** | **0.68 [0.48, 0.94]*** |
|  |  |  |
| PTSD Symptoms | 1.07 [0.87, 1.03] | 1.02 [0.81, 1.28] |
| Personalized (vs. placebo) | 0.82 [0.51, 1.34] | 0.78 [0.45, 1.37] |
| Non-Personalized (vs. placebo) | 1.01 [0.62, 1.64] | 1.00 [ 0.58, 1.73] |
| Placebo | Ref | Ref |
| Study Day | 1.00 [0.94, 1.05] | 1.02 [0.96, 1.08] |
| Female (vs. male) | **0.59 [0.37, 0.93]*** | 0.68 [0.40, 1.15] |
| *Note.* Model results from separate Logistic mixed effects models with the primary predictor and daily heavy drinking outcome, adjusting for ABM condition (personalized, non-personalized, placebo), study day, and sex.  Significance is indicated after confidence intervals: *+p_raw_<.10*, ***p_raw_<.05**, ****p_raw_<.01**.  BH-FDR corrected p-values (applied to threat sensitivity models): †p_BH-FDR_<.10, ††p_BH-FDR_<.05. | | |
